# Metal Nanoparticles Produced Using Autotrophs and Their Bioproducts: A Comparative Overview between Photosynthesizing Taxonomic Groups

**DOI:** 10.1021/acsomega.4c11418

**Published:** 2025-04-01

**Authors:** Mateus Fernandes Oliveira, Leonardo César Moraes, Cleber Cunha Figueredo

**Affiliations:** Departamento de Botânica, Instituto de Ciências Biológicas, Universidade Federal de Minas Gerais, Av. Antônio Carlos, 6627, Pampulha, Belo Horizonte, Minas Gerais 31270-901, Brazil

## Abstract

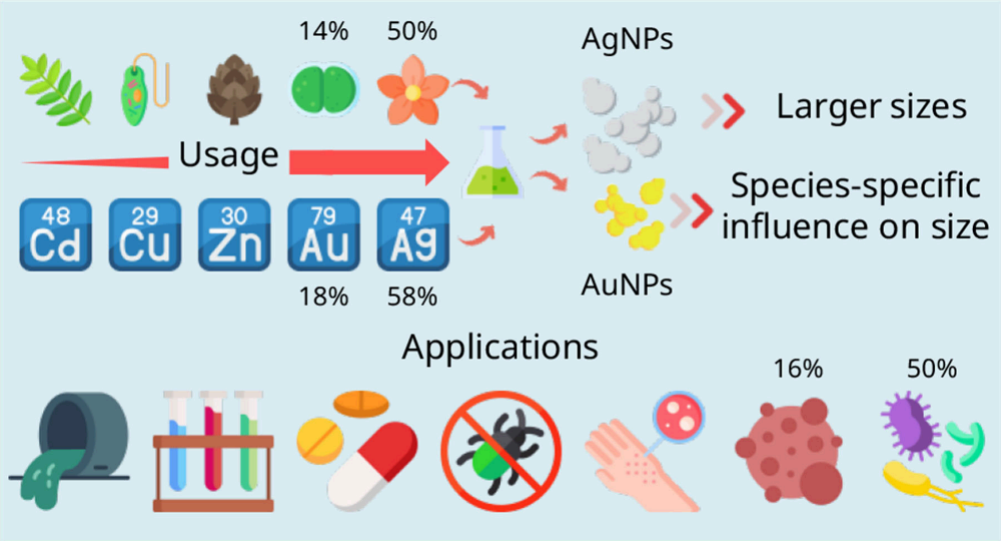

Metal nanoparticles (MNPs) exhibit unique properties influenced by their size, shape, and dispersion uniformity. They can be synthesized via chemical methods or green synthesis, commonly using plant or microorganism extracts as reducing and stabilizing agents. This eco-friendly approach is valued, but the literature is unclear about which taxonomic groups should be targeted to obtain certain types of MNPs. Given the ongoing growth of research in this area, this study offers a comparative overview that helps identify patterns and gaps in the current knowledge. This study reviewed 485 articles, describing 652 monometallic and 10 bimetallic nanoparticles synthesized using photosynthesizing organisms’ extracts. Angiosperms and cyanobacteria were the most utilized groups. Silver and gold nanoparticles were the most studied MNPs. Gold nanoparticles’ size varied with taxonomic groups, but they were smaller than the silver nanoparticles synthesized by the same group. Antimicrobial activity was the most common application, highlighting the potential of green-synthesized MNPs. This study provides valuable insights for optimizing sustainable nanoparticle production since knowledge about the specificities of different photosynthesizing taxa can be useful for directing efforts and enhancing the efficiency and precision of green-synthesized MNPs.

## 1 Introduction

Nanoparticles (NPs) are structures ranging from 1 to 100 nm, and they exhibit unique physical and chemical properties influenced by their size, shape, and dispersion uniformity.^1−3^ Smaller NPs are of particular interest due to their higher surface atom exposure, which enhances chemical reactivity and their ability to infiltrate cells and tissues.^4^ The shape of NPs is relevant because it influences their efficiency in many different processes.^5−9^ Dispersion is also critical since NPs with more uniform dispersion are more efficient, increase predictability of results, and have consistent performance, while promoting the stability of colloidal suspensions by preventing aggregation and sedimentation.^10,11^

The most common NPs are made of carbon,^12^ metals,^13^ lipids,^14^ or polymers,^15^ offering a wide range of possibilities for different applications. Despite this great variety in the chemical composition of NPs, metallic ones (MNPs) stand out for their versatility and wide range of applications.^4,16^ Composed especially of noble metals, such as gold,^17,18^ silver,^19,20^ and platinum,^21^ MNPs have some particular characteristics that are pivotal in several domains, including relevant technological innovations and scientific advancements.^13^ In medicine, MNPs increase diagnostic precision,^22^ control drug delivery,^23^ and allow for earlier identification of diseases.^24^ The more recent applications of MNPs include cosmetics production,^25^ as a facilitating agent for various catalytic processes,^26^ and in energy technologies by enhancing the efficiency of solar cells.^27^

Metal NPs can be synthesized through conventional chemical procedures^28,29^ or environmentally friendly green methodologies.^30,31^ Traditional MNP synthesis typically involves chemical reducing agents, such as sodium borohydride, which donate electrons to metal salts, reducing them to NPs under high temperature or pressure conditions.^28,29^ In green synthesis, biomolecules such as flavonoids, phenols, or NADPH act as reducing agents for metal ions,^32,33^ forming NPs under mild conditions using nontoxic solvents and generating biodegradable byproducts.^30,31,34,35^ Advancing green NP synthesis is crucial, as it offers an environmentally friendly alternative and unlocks new possibilities in medicine, environmental solutions, and energy innovations, promising to be a sustainable trajectory in nanotechnology.^31^

The biological green synthesis of MNPs involves both heterotrophs and autotrophs,^31^ but the latter are particularly noteworthy due to their role as primary producers and their relatively easier cultivation and maintenance.^36^ Despite numerous studies performed with many different taxa—such as cyanobacteria,^37^ microalgae,^38^ macroalgae,^39^ bryophytes,^40^ and vascular plants^41^—there is a lack of comprehensive comparative analysis of the characteristics of MNPs produced by all these organisms. Only a few studies have made comparisons, and they were almost always focused on related species or phyla (e.g., AgNPs^42^). There are also some review articles that aggregate data on MNP properties across taxonomic groups, but without detailed comparisons.^43^ Only a recent study was based on a comparative analysis, identifying the relationships between cyanobacteria and microalgae species and the characteristics of silver NPs green-synthesized by these microorganisms.^44^

To optimize NP synthesis and show the potential of green synthesis in producing chemically valuable and applicable MNPs, this study compared MNPs synthesized by several photosynthetic organisms, focusing on the choices of organisms and metals by researchers as well as NP size, shape, and dispersion. With over 460000 described species,^45,46^ the diversity of photosynthetic organisms presents immense potential for NP generation. However, this diversity also poses challenges in identifying the most suitable organisms and metals for synthesis. By analyzing the existing literature, we aim to identify variations in NP characteristics across taxonomic groups and to uncover gaps in the application of specific metals, taxonomic groups, and species. Understanding these patterns and differences will help refine the selection process for organisms and metals to be used in MNP synthesis, enabling more targeted and efficient synthesis methods, reducing costs, advancing scientific knowledge, and supporting technological innovation and environmental sustainability.

## 2 Materials and Methods

### 2.1 Literature Review

We used the book by Srivastava and Bhargava^31^ as a starting point for our review. This book is relevant because it presents the latest advancements in green NP synthesis, addresses techniques for characterizing biosynthesized NPs, and evaluates both the applications and potential toxicity of these materials. Additionally, this book features specific chapters on green synthesis of NPs through major groups of photosynthetic organisms (e.g., microalgae, macroalgae, bryophytes, pteridophytes, gymnosperms, and angiosperms). Thus, all the articles gathered within these chapters were evaluated together with our own new survey of the literature.

For surveying the recent literature focused on green synthesis of MNPs, we performed an assessment using records obtained from Google Scholar by crossing the terms “green synthesis”*”nanoparticles” with the groups of photosynthetic organisms cited in Srivastava and Bhargava.^31^ Although many types of studies were obtained, we considered only scientific articles that focused on NPs green synthesized by photosynthetic organisms and their bioproducts, covering the period from January 2022 to May 2024. Although this research builds upon the foundational work presented in the book by Srivastava and Bhargava,^31^ our approach is not a complementary survey, since it involves analyzing the entire database in an innovative way.

We compiled 542 articles (Supporting Information), but only 485 were considered for extracting information for the analyses. Given that the definition of NPs encompasses structures smaller than 100 nm,^47^ all articles reporting structures larger than 100 nm were excluded from this review. Furthermore, studies with no information about nanoparticle size were also excluded to avoid the possibility of considering larger particles.

### 2.2 Data Compilation

Each article was used to access data on the taxonomic group, genus, and species of the focal organism studied (Supporting Information). Additionally, for vascular plants, we also documented the plant parts (e.g., leaves, roots, stems, and reproductive structures) used for the extract preparation for NP production. A detailed compilation of information was also performed in relation to the NPs, reporting the metal used for MNP synthesis, along with its size, shape, and dispersion characteristics (Supporting Information). In cases where this information was not directly available in the articles, we noted it as “undetermined”. Regarding dispersion, if the articles did not explicitly address this critical nanoparticle characteristic, we examined whether histograms or the Polydispersity Index (PDI) of nanoparticle size were provided. Based on our observations, we classified them as monodisperse or polydisperse and denoted them with an asterisk in the Supporting Information.

To facilitate our understanding of nanoparticle uses, we simplified applications into eight main categories: (I) antimicrobial capacity; (II) antioxidant and anti-inflammatory activities; (III) catalytic activity; (IV) anticancer and cytotoxic activities; (V) insecticidal and acaricidal activities; (VI) pollutant removal and/or detection; (VII) disease treatments; (VIII) other applications. These categories encompass a broad spectrum of potential uses for NPs across scientific and industrial applications. The specific use for each compiled nanoparticle article can be found in the Supporting Information for verification.

### 2.3 Statistical Analysis

We investigated the influence of photosynthetic organisms on nanoparticle size, with a particular focus on the two most commonly used metals. We included only the taxonomic groups with at least three nanoparticle descriptions for each metal, excluding groups that did not meet this criterion from the analyses. To ensure normality and homogeneity of variances, data were previously transformed into log (*x* + 1). Before conducting the statistical analysis, we first performed the Shapiro–Wilk test to assess the normality of the data, which indicated that the data did not follow a normal distribution (*p* < 0.05). Therefore, we opted for a nonparametric approach, specifically the Kruskal–Wallis test. In case of significant results from this test, we performed Dunn’s post hoc tests to identify which specific groups differed from each other. These analyses were performed using R software version 4.4.2.^48^

In addition to the above-mentioned methods, we conducted an analysis to determine whether the two most commonly used metals produce NPs of significantly different sizes within each group of photosynthetic organisms. For this analysis, we selected only the taxonomic groups that had at least five nanoparticle size descriptions for both of the most commonly used metals in the green synthesis of NPs. Prior to the statistical analyses, we assessed the normality of the data using the Shapiro–Wilk test, which revealed that the data did not follow a normal distribution (*p* < 0.05). Consequently, we employed a nonparametric approach, specifically the Mann–Whitney *U* test. This test was applied separately to each taxonomic group. All analyses were performed using R software version 4.4.2.^48^

## 3 Results

### 3.1 Overview of MNPs: Which Metals and Organisms Are Utilized?

**Figure 1:**
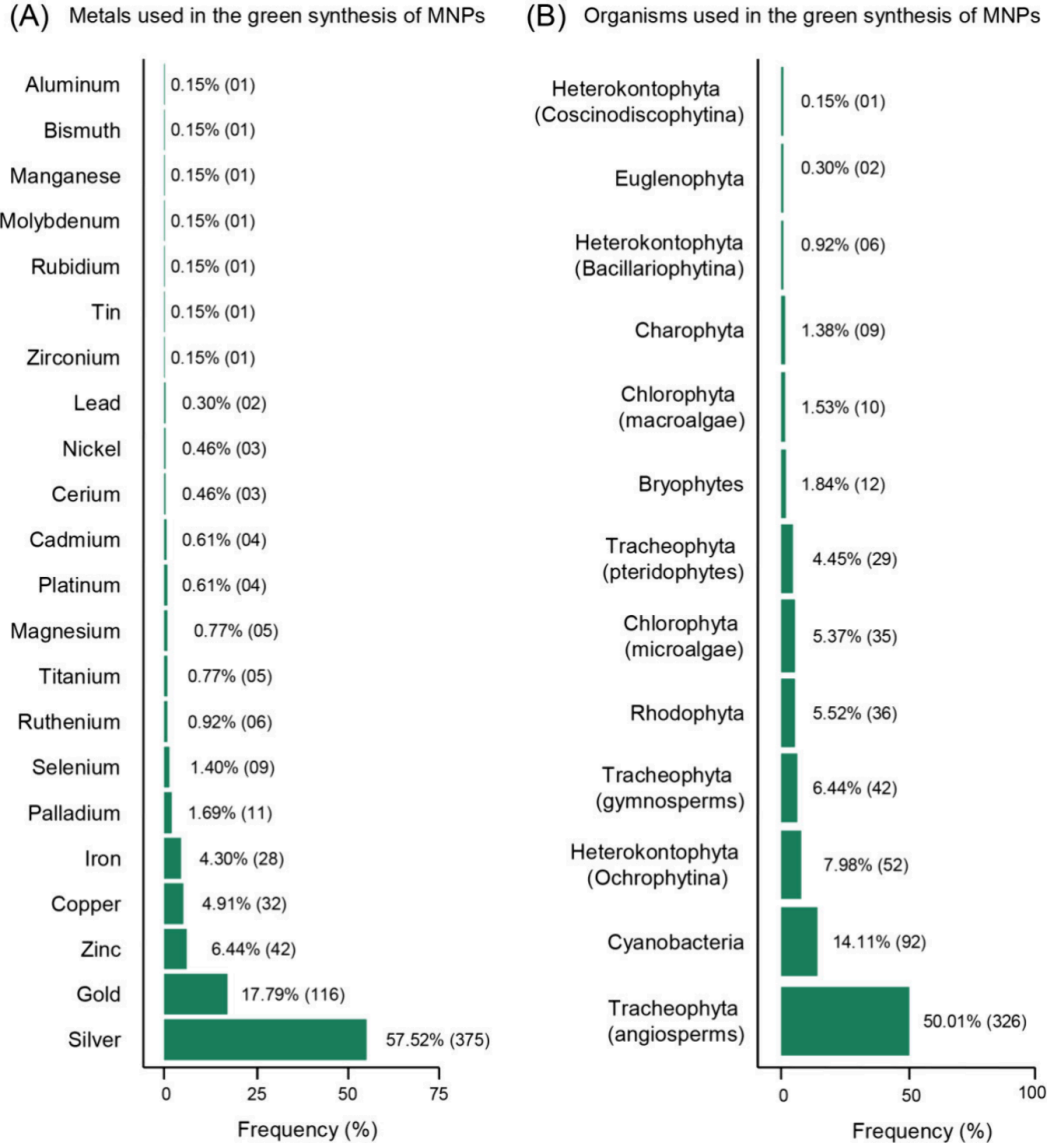
Overview of metals and taxonomic groups used in the green synthesis of monometal NPs. (A) The percentage distribution of the used metals, with silver being the most common. (B) The frequency of taxonomic groups utilized in the green synthesis of MNPs, emphasizing that angiosperms are the most frequently represented group in the reviewed literature. The total number of compiled descriptions is indicated in parentheses.

The analyses of the 485 compiled articles yielded 652 descriptions of monometal NPs synthesized from extracts of photosynthetic organisms and only ten descriptions for bimetallic MNPs (Supporting Information). Thus, the MNPs were composed of one or two of 22 different metals: aluminum (Al), bismuth (Bi), cadmium (Cd), cerium (Ce), copper (Cu), gold (Au), iron (Fe), lead (Pb), magnesium (Mg), manganese (Mn), molybdenum (Mo), nickel (Ni), palladium (Pd), platinum (Pt), rubidium (Rb), ruthenium (Ru), silver (Ag), selenium (Se), tin (Sn), titanium (Ti), zinc (Zn), and zirconium (Zr). The most frequently used metal in the green synthesis of NPs is Ag (57.52%), followed by Au (17.79%), Zn (6.44%), and Cu (4.91%), with the 18 remaining 18 metals collectively accounting for only 13.34% of the MNPs reported in the compiled studies (Figure 1A).

A high diversity of photosynthetic organisms was used for the production of MNPs (Supporting Information). For didactic purposes, the taxonomy was reviewed based on current systems (Algaebase.org and Tropicos.org), and the taxa were divided into 13 groups as follows: Cyanobacteria; Chlorophyta subdivided into microalgae (Chlorophyceae and Trebouxiophyceae) and macroalgae (Ulvophyceae); Heterokontophyta subdivided into the subphyla Bacillariophytina (Bacillariophyceae and Mediophyceae), Coscinodiscophytina (Coscinodiscophyceae), and Ochrophytina (Eustigmatophyceae and Phaeophyceae); Euglenophyta; Rhodophyta; Charophyta; bryophytes (including liverworts, hornworts, and mosses); Tracheophyta subdivided into pteridophytes (lycophytes and ferns), gymnosperms, and angiosperms. The frequency of taxonomic groups used in green synthesis of MNPs varies (Figure 1B), with the most widely utilized being angiosperms (50.01%), followed by Cyanobacteria (14.11%) and Heterokontophyta - Ochrophytina (7.98%). In contrast, the less utilized groups are Euglenophyta (0.30%) and Heterokontophyta - Coscinodiscophytina (0.15%).

Some groups exhibited relatively high biodiversity in nanoparticle production, such as angiosperms, where 179 distinct plant genera were utilized for the green synthesis of MNPs (Table 1). Considering all the taxonomic groups, the genus *Sargassum* C. Agardh (Heterokontophyta - Ochrophytina) was the most utilized (2.8% of the compiled descriptions), with 11 species being applied (Table 1). The other most utilized genera were *Spirulina* Turpin ex Gomont (Cyanobacteria; 1.5% of the compiled descriptions) and *Zingiber* Boehm. (Tracheophyta - angiosperms; 1.4% of the compiled descriptions). In contrast, the less studied groups had only a single species as the focus of research, namely *Euglena gracilis* G.A. Klebs in the phylum Euglenophyta,^49^ and *Eupyxidicula turris* (Greville) S. Blanco & C.E. Wetzel among the Heterokontophyta - Coscinodiscophyceae.^50^

**Table 1:** Most Used Organisms for the Green Synthesis of Monometal NPs by Taxonomic Group^a^

Taxonomic group	Genus	Species	References
Cyanobacteria (**32**)	*Spirulina*	*S. platensis*	(51−53)
*Chlorophyta - Microalgae* (**17**)	*Chlorella*	*C. vulgaris*	(54−59)
		*C. minutissima*	
Chlorophyta - Macroalgae (**07**)	*Caulerpa*	*C. serrulata*	(60, 61)
		*C. racemosa*	
Charophyta (**04**)	*Cosmarium*	*C. impressulum*	(44, 49, 62)
		*C. punctulatum*	
Euglenophyta (**01**)	*Euglena*	*E. gracilis*	(49)
Rhodophyta (**21**)	*Gracilaria*	*G. birdiae*	(63−66)
		*G. dura*	
		*G. edulis*	
Heterokontophyta - Ochrophytina (**19**)	*Sargassum*	*S. acinarium*	(67−85)
		*S. boveanum*	
		*S. incisifolium*	
		*S. glaucescens*	
		*S. muticum*	
		*S. myriocystum*	
		*S. plagiophyllum*	
		*S. swartzii*	
		*S. tenerrimum*	
		*S. wightii*	
Heterokontophyta - Bacillariophytina (**05**)	*Navicula*	*N. cincta*	(86)
Heterokontophyta - Coscinodiscophytina (**01**)	*Eupyxidicula*	*E. turris*	(50)
Bryophytes (**10**)	*Bryum*	*B. argenteum*	(87−89)
		*B. medianum*	
		*B. salakense*	
Tracheophyta - pteridophytes (**18**)	*Selaginella*	*S. bryopteris*	(90−93)
		*S. convolute*	
		*S. myosurus*	
Tracheophyta - gymnosperms (**14**)	*Pinus*	*P. densiflora*	(94−101)
		*P. eldarica*	
		*P. merkusii*	
		*P. roxburghii*	
		*P. thunbergii*	
		*P. wallichiana*	
Tracheophyta - angiosperms (**179**)	*Zingiber*	*Z. officinale*	(102−106)

a The total number of compiled genera per group is indicated in parentheses.

**Figure 2:**
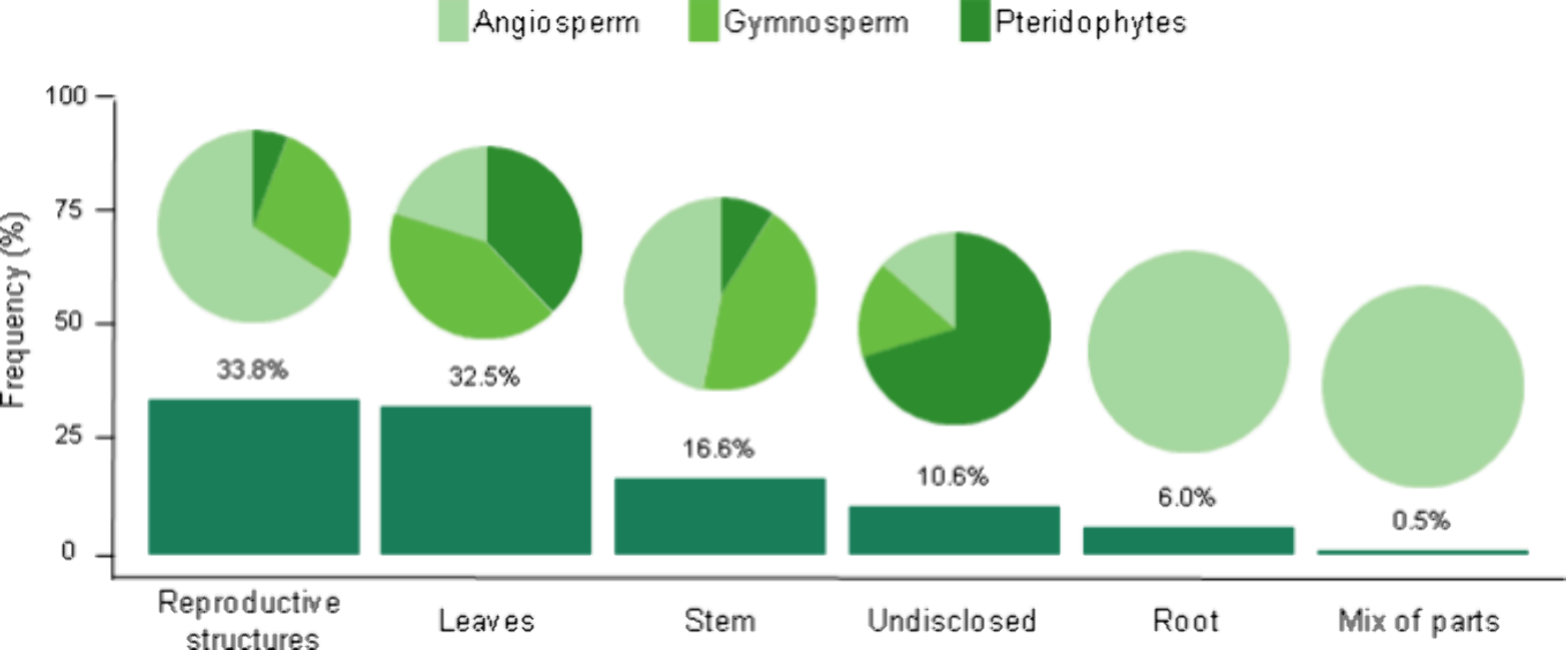
Frequency (%) of usage of different parts of tracheophytes (pteridophytes, gymnosperms, and angiosperms) in the green synthesis of MNPs. The pie charts illustrate the proportional contribution of each plant group to the total usage of each plant part.

Due to the structural complexity of organisms within Tracheophyta, the plant parts (e.g., leaves, roots, stems, reproductive structures) are commonly used separately for extract preparation in MNP production. Thus, detailed data for each part were compiled separately. Reproductive structures (spores, flowers, fruits, or seeds) were the most frequently used plant parts, accounting for 33.8% of the total compiled descriptions, with the majority of studies focusing on angiosperms (Figure 2). Leaves followed closely (32.5%), although most studies utilized leaf extracts from pteridophytes and gymnosperms. Although, in order to better organize the data set, the synthesis of MNPs using plant extracts was evaluated separately for parts of these organisms, it was difficult to do this for pteridophytes. This occurred because studies focused on these organisms commonly do not clearly mention which exact part of the plant was used (Figure 2).

### 3.2 Patterns in Monometal Nanoparticle Synthesis by Photosynthetic Groups

**Figure 3:**
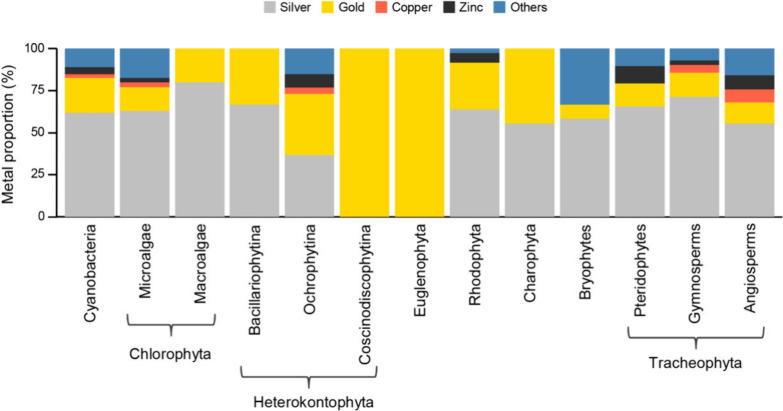
Proportion of metals used by photosynthetic groups in the green synthesis of monometal NPs.

Once Ag is the metal most frequently used in the green synthesis of monometallic NPs using extracts of autotrophs, it is observed that this element is also the most used for almost all taxonomic groups. The unique metal that was used for green synthesis with all of the taxonomic groups was Au (Figure 3). Since Heterokontophyta - Coscinodiscophyceae and Euglenophyta were tested exclusively for AuNPs synthesis, they obviously exhibited Au as the most explored metal. The Chlorophyta - macroalgae group, Heterokontophyta - Bacillariophytina group, and Charophyta were explored for only AuNP and AgNP biosynthesis. Both Au and Ag were utilized in similar proportions for MNP production within the Heterokontophyta - Ochrophytina group, but this group was also tested for other metals (Figure 3). The other photosynthetic groups had 50% of the MNPs produced using Ag (Figure 3). Additionally, only Cyanobacteria, Chlorophyta (microalgae), Heterokontophyta - Ochrophytina, gymnosperms, and angiosperms had Ag, Au, Zn, and Cu explored for nanoparticle production. The less studied metals (mentioned as the “others” group) for green synthesis of MNPs were tested in bryophytes more than in the other autotrophic groups (Figure 3).

**Figure 4:**
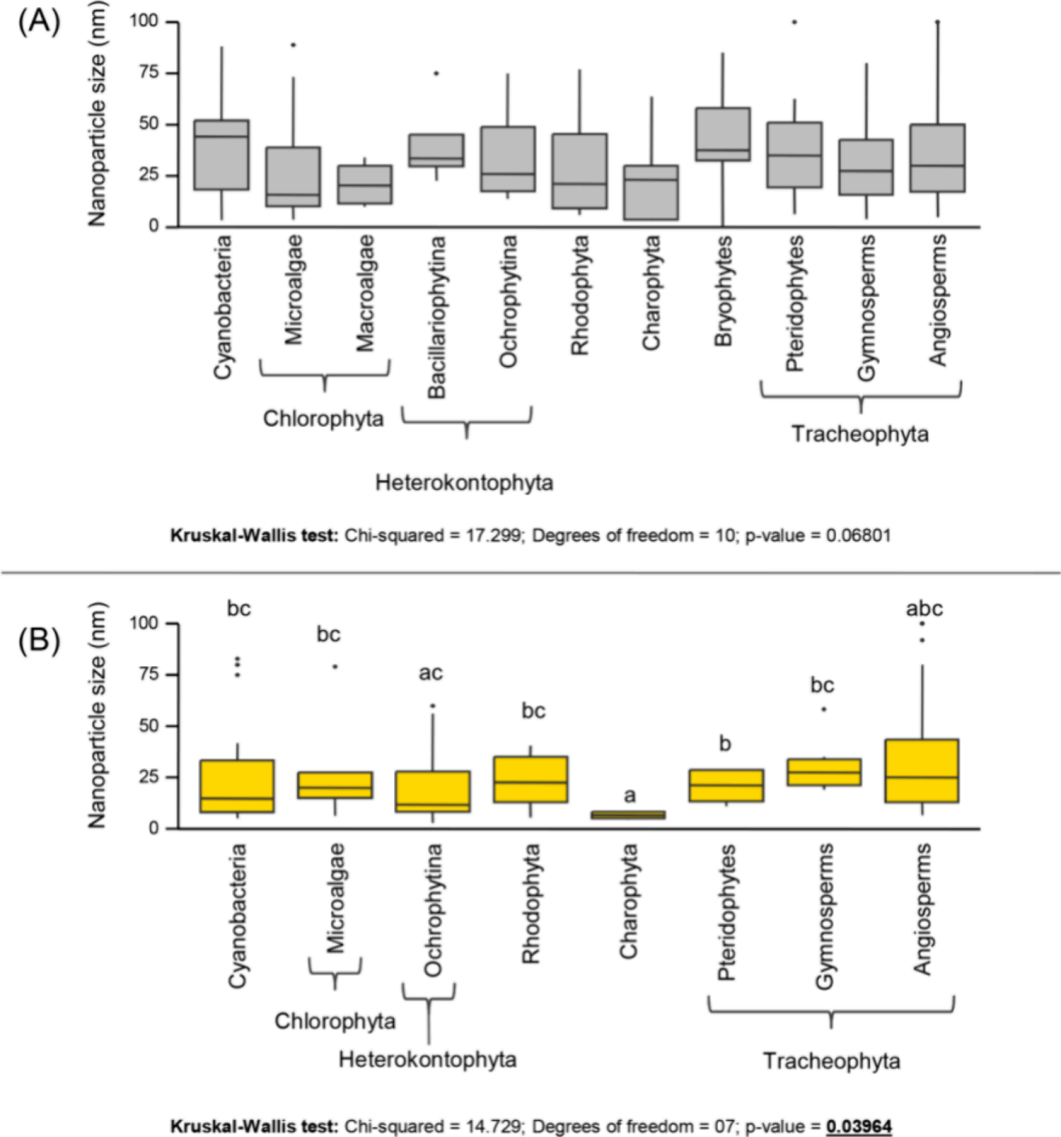
Monometallic nanoparticle size across the photosynthetic group: (A) silver MNPs; (B) gold MNPs. Lines in boxes are medians, ends of boxes show quartiles, and black dots are outliers. Significant values for the Kruskal–Wallis test are in bold and highlighted.

The comparisons among the MNP sizes according to different taxonomical groups were focused on the metals most commonly employed in green synthesis (Ag and Au). The Kruskal–Wallis test revealed no significant differences in the size of AgNPs across taxonomic groups (*p* = 0.068; Figure 4). However, the sizes of AuNPs were significantly different among the taxonomic groups (*p* = 0.039). Notably, Charophyta produced the smallest NPs, exhibiting a significant difference from the majority of other groups (Figure 4). Additionally, the average size of AuNPs produced by Heterokontophyta - Ochrophytina differed from those produced by pteridophytes.

**Figure 5:**
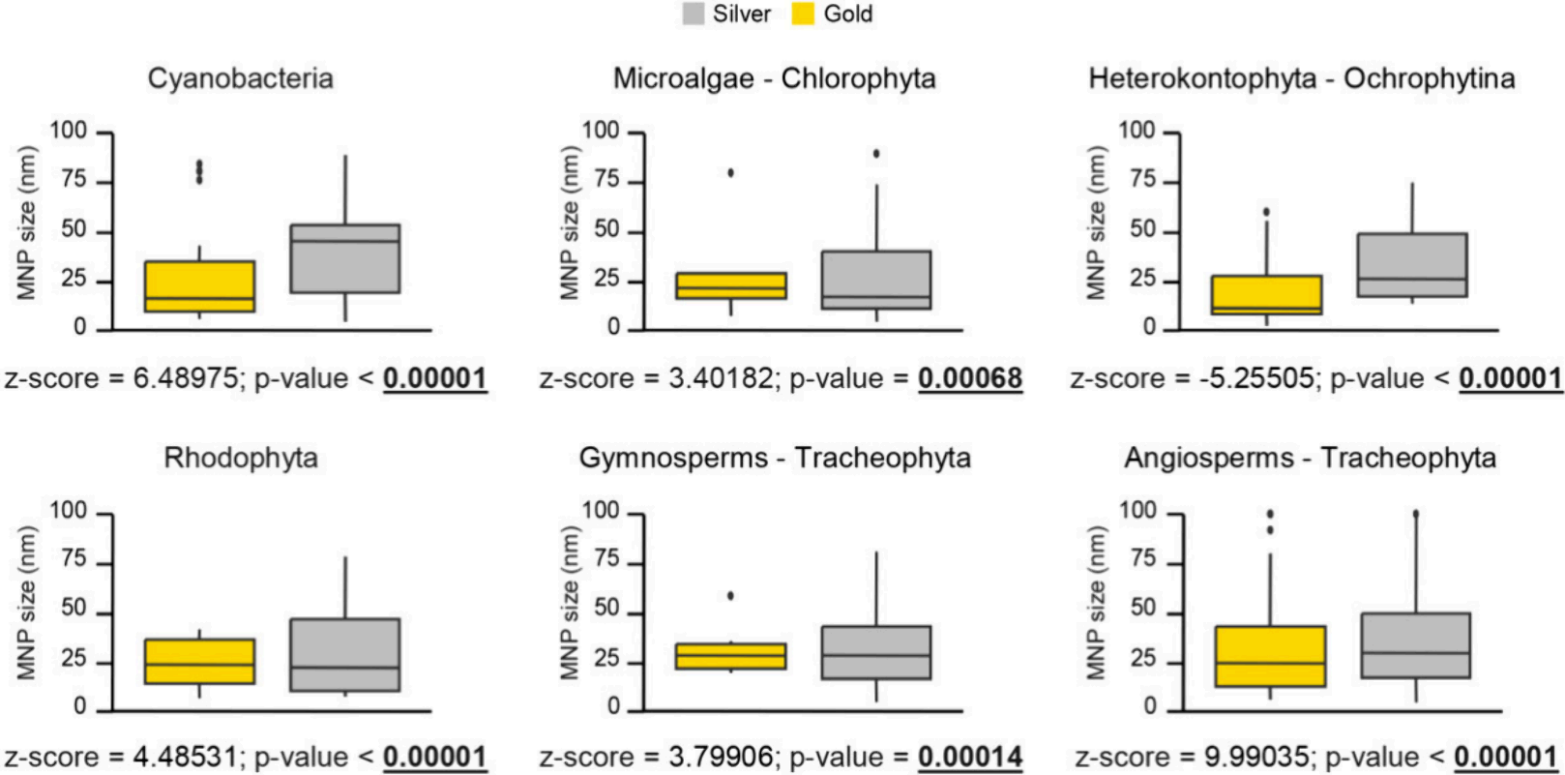
Comparison of the average nanoparticle sizes of gold and silver across different taxonomic groups. The lines within the boxes represent the medians, the ends of the boxes indicate the quartiles, and the black dots denote outliers. Statistically significant values from the Mann–Whitney *U* test are highlighted in bold.

When the sizes of AgNPs and AuNPs produced within each taxonomic group were compared, some significant differences were observed (Figure 5). The Mann–Whitney *U* test revealed that Cyanobacteria, Chlorophyta - microalgae, Heterokontophyta - Ochrophytina, Rhodophyta, gymnosperms, and angiosperms produce NPs of differing sizes for silver and gold, with silver NPs being larger (Figure 5). The remaining taxonomic groups have an insufficient number of size descriptions to allow these investigations to be carried out.

Among the MNPs produced using the four most commonly used metals (Ag, Au, Zn, and Cu), the smallest NPs were AuNPs synthesized by Charophyta (6.53 ± 2.07 nm; Table 2). The smallest AgNPs (20.82 ± 10.08 nm) were produced by macroalgae Chlorophyta, while the smallest ZnNPs (20.00 nm) were synthesized by gymnosperms, and the smallest CuNPs (13.78 ± 9.73 nm) were produced by Heterokontophyta - Ochrophytina. In contrast, the largest MNPs included CuNPs from Chlorophyta - Microalgae (70.78 nm), followed by ZnNPs from Rhodophyta, AgNPs from bryophytes, and AuNPs from angiosperms (Table 2).

**Table 2:** Mean Values of Monometal NP Size (nm) of the Four Most Frequent Types Synthesized Using Extracts from Photosynthetic Organisms^a^

	MNP average size (mean ± standard deviation)			
Taxonomic group	Silver (nm)	Gold (nm)	Zinc (nm)	Copper (nm)
Cyanobacteria	39.59 ± 21.19	28.40 ± 27.75	40.18 ± 19.11	47.50 ± 31.82
Chlorophyta - microalgae	24.94 ± 19.46	29.50 ± 28.75	33.40*	70.78*
Chlorophyta - macroalgae	20.82 ± 10.08	29.20 ± 18.66		
Charophyta	24.76 ± 24.63	6.53 ± 2.07		
Euglenophyta		11.20 ± 0.14		
Rhodophyta	28.40 ± 22.26	23.47 ± 12.93	50.02 ± 47.67	
Heterokontophyta - Ochrophytina	33.83 ± 19.87	18.77 ± 16.64	37.53 ± 13.67	13.78 ± 9.73
Heterokontophyta – Bacillariophytina	41.13 ± 23.20	15.50 ± 9.19		
Heterokontophyta - Coscinodiscophytina		20.00*		
Bryophytes	47.64 ± 20.79	70.00*		
Tracheophyta – pteridophytes	37.55 ± 24.14	20.50 ± 9.32	43.53 ± 7.92	
Tracheophyta - gymnosperms	31.75 ± 19.80	31.20 ± 14.55	20.00*	22.50 ± 7.07
Tracheophyta - angiosperms	35.03 ± 23.24	33.87 ± 28.54	42.49 ± 22.48	23.54 ± 15.84

a Unique values are indicated with an asterisk, highlighting instances where only one study reported on the formation of MNPs using the specified taxonomic group and metal.

**Figure 6:**
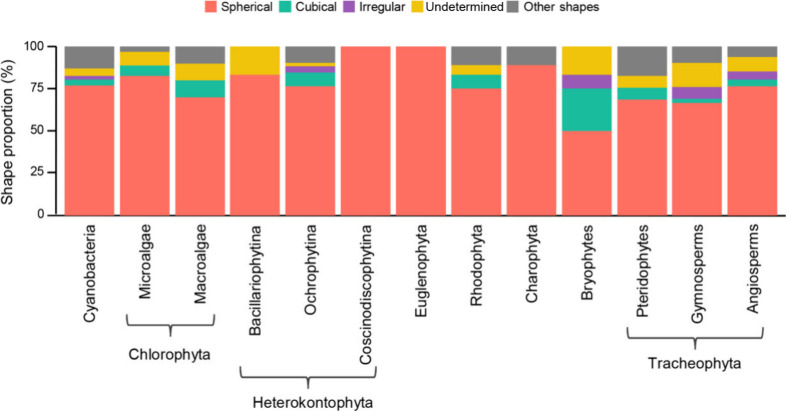
Proportional distribution of monometallic nanoparticle shapes by taxonomic group.

Regarding the shape of MNPs, the spherical ones predominated across all taxonomic groups, with 50% of the descriptions for each group reporting this shape, except for bryophytes (Figure 6). Cubical MNPs were the second most common type of MNPs, although their frequency was lower compared to studies that did not specify MNP shapes in some taxonomic groups. Notably, only Cyanobacteria, Heterokontophyta - Ochrophytina, bryophytes, gymnosperms, and angiosperms were reported to produce all three shapes surveyed (spherical, cubical, and irregular; Figure 6). Many studies lacked information on MNP shape, resulting in a relevant category of indeterminate shapes, especially for bryophytes, gymnosperms, Chlorophyta (both microalgae and macroalgae), and Heterokontophyta - Bacillariophytina.

**Figure 7:**
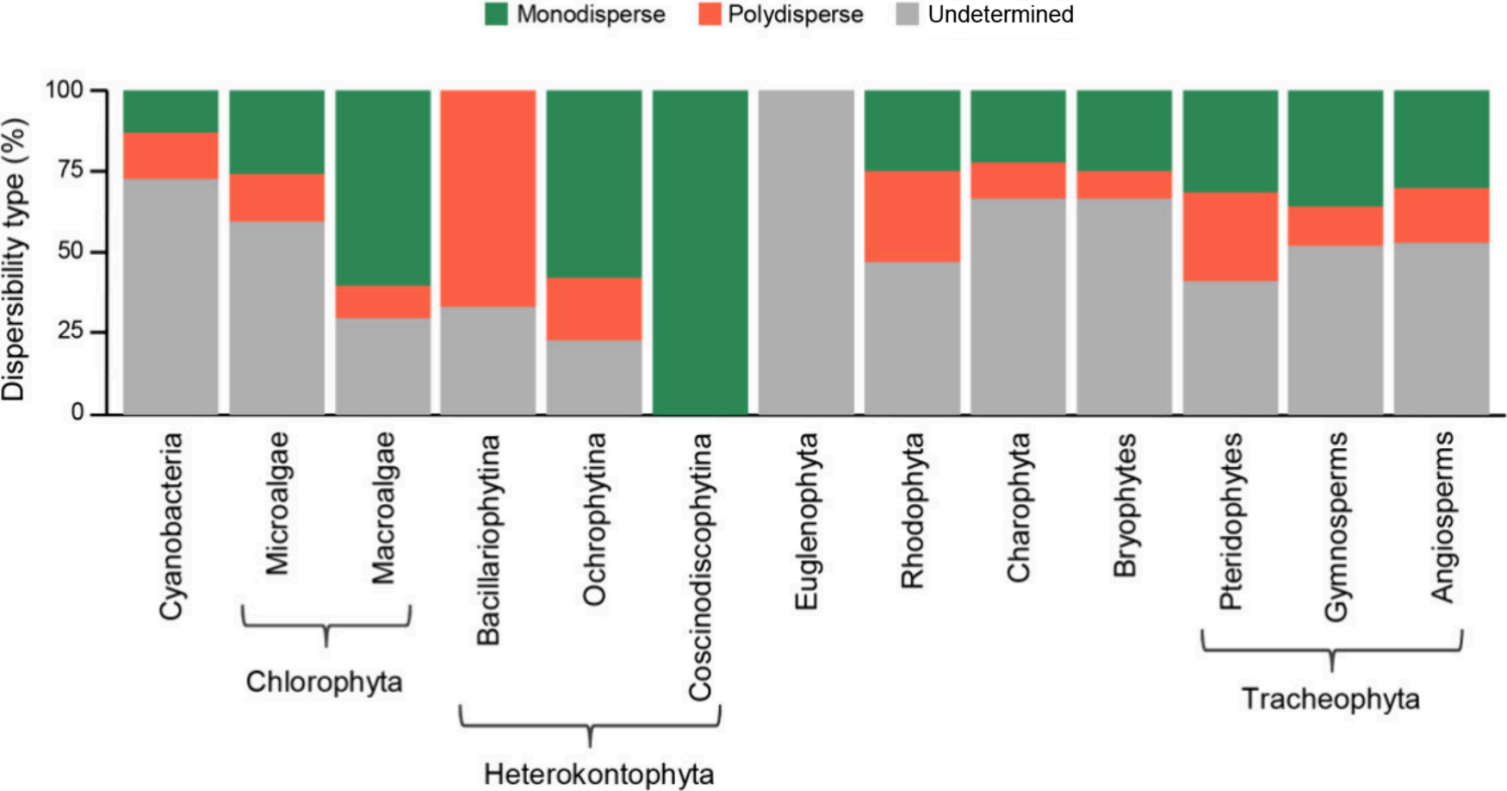
Dispersity of monometal NPs among photosynthetic groups utilized in green synthesis.

The scarcity of information regarding nanoparticle dispersity is particularly notable, with 53.4% of the 652 descriptions of monometallic NPs failing to mention this property. Specifically for the most studied taxonomic groups (cyanobacteria, microalgae: Chlorophyta, Charophyta, bryophytes, gymnosperms, and angiosperms), 50% of the compiled descriptions do not provide details on NP dispersity (Figure 7). The information for the less studied groups obviously does not allow establishing a pattern. For Euglenophyta, which has only one published study on NPs, no information about NP dispersity was revealed. Conversely, the sole study on Coscinodiscophytina - Heterokontophyta indicates that the NPs were monodisperse. Additionally, more than 50% of the nanoparticles synthesized by macroalgae - Chlorophyta and Ochrophytina - Heterokontophyta were monodisperse (Figure 7).

Regarding applications, only 28% of the studies were descriptive of the capacity of autotrophs to synthesize MNPs, with no test for uses. Most of the studies (72%) have also focused on performing tests to search for an application for the MNPs synthesized. Antimicrobial activity was the main focus (Table 3), corresponding to 49.5% of the applications tested. Different photosynthetic groups and different metals, such as SeNPs from Cyanobacteria,^107^ AgNPs from bryophytes,^89^ and CeNPs from angiosperms,^108^ were employed for this purpose. The second largest proportion of the studies (16.2%) was concentrated on anticancer and cytotoxic activities, especially using NPs produced with gymnosperms and angiosperms.^101,109,110^ Notably, many studies with applications tended to focus on multiple applications simultaneously, resulting in the number of applications (699) exceeding the number of nanoparticle descriptions (652) and compiled articles (485).

**Table 3:** Applications of the Synthesized MNPs by Photosynthetic Organism^a^

	Applications: Total number of nanoparticle descriptions per application							
Taxonomic group	AA	AAA	CA	ACA	IAA	PRD	DTA	OA
Cyanobacteria	5	5	3	8	1	6	5	1
Chlorophyta - microalgae	15	1	2		1	3		
Chlorophyta - macroalgae	6		2					
Charophyta	1					1		
Euglenophyta								
Rhodophyta	19	2		7	3	1		1
Heterokontophyta – Ochrophytina	21	3	4	8	3	6	2	1
Heterokontophyta - Bacillariophytina	6					1		
Heterokontophyta - Coscinodiscophytina								
Bryophytes	7	2		2		1		1
Tracheophyta - pteridophytes	13	8	1	5	2	1	5	2
Tracheophyta - gymnosperms	25	7	3	16	1	2		
Tracheophyta - angiosperms	203	60	27	67	13	20	16	10
**Total**	**346**	**88**	**42**	**113**	**24**	**42**	**28**	**16**

a Abbreviations: AA, antimicrobial activity; AAA, antioxidant and anti-inflammatory activities; CA, catalytic activity; ACA, anticancer and cytotoxic activities; IAA, insecticidal and acaricidal activities; PRD, pollutant removal and/or detection; DTA, disease treatment activities; OA, other applications.

### 3.3 What Do We Know About the Green Synthesis of Bimetallic NPs?

Bimetal nanoparticles optimize the plasmon absorption band’s energy, offering multifunctionality for biosensors and distinct optical, electronic, thermal, and catalytic properties compared to monometal NPs.^111^ Despite their potential, green syntheses using photoautotrophs are rare, representing only 1.5% of the compiled studies. These NPs were predominantly produced using angiosperms (Table 4) and their morphological traits were also well characterized, being predominantly spherical and smaller than 50 nm. On the other hand, their dispersibility was investigated in only two studies (e.g., Pd–Pt NPs,^112^ Au–Ag NPs^113^), both of which report these NPs as monodisperse. Copper and gold were the metals most commonly used in combination for the formation of bimetallic NPs (Table 4). Many studies did not test applications for these NPs,^68,112,117^ while other authors explored their applications in antibacterial,^113,118,120^ catalytic,^115,116^ anticancer,^118^ photocatalytic dye degradation,^118,121^ and cytotoxicity tests.^119^

**Table 4:** Green Synthesized Bimetal NPs Using Photosynthetic Organisms and Their Characteristics

Taxonomic group	Metals	Shape	Mean size (nm)	Dispersibility	References
Cyanobacteria	Au–Ag	Spherical	21.00		(114)
Tracheophyta - angiosperms	Pd–Pt		4.73	Monodisperse	(112)
Tracheophyta - angiosperms	Au–Ag	Hexagonal	90.00	Monodisperse	(113)
Tracheophyta - angiosperms	Pd–Cu		40.00		(115)
Tracheophyta - angiosperms	Pd–Fe		39.00		(116)
Chlorophyta - microalgae	Cu–Fe	Spherical	24.00		(117)
Tracheophyta - angiosperms	Ag–Cu	Spherical	69.80		(118)
Tracheophyta - angiosperms	Ag–Se	Spherical	21.00		(119)
Tracheophyta - angiosperms	Ag–Ti	Irregular	30.00		(120)
Tracheophyta - angiosperms	Cu–Fe	Hexagonal	23.93		(121)

## 4 Discussion

### 4.1 Metals Used in Nanoparticle Green Synthesis

The wider use of Ag and Au for green synthesis of MNPs is probably related to their unique characteristics, with silver being the most commonly used metal because of several factors that make it particularly important in the commercial sphere of nanotechnology and general studies worldwide.^122^ In fact, silver is particularly important due to its high electrical and thermal conductivity, low contact resistance,^123^ and greater abundance in the Earth’s crust when compared to gold,^124^ making it more cost-effective. Silver NPs exhibit versatile modes of action, including electron activation of functional groups in ligands^62^ and the release of Ag^+^ ions, which can disrupt electron carrier chains and affect biomolecules like plasma membranes, genetic material, and proteins through reactive oxygen species (ROS).^125^ Although toxic at high concentrations,^126^ silver’s biocompatibility in controlled amounts allows its application in various biomedical fields,^127^ particularly for its antimicrobial^128,129^ and anticancer properties.^130,131^

Although gold was the second most used metal in green nanoparticle synthesis, it was used three times less than silver. However, Au offers distinct advantages, such as being chemically inert in bulk form but highly active as NPs.^132^ Gold NPs (AuNPs) are versatile, generally nontoxic, and have unique properties like substantial surface modifiability, biocompatibility, and quenching efficiency,^17,133^ making them advanced materials for tumor treatment.^134^ Their surface can be easily functionalized with various chemical groups and biomolecules,^135^ enabling tailored applications in diagnostics and targeted therapies.^136,137^ Despite higher costs, these benefits explain their use in less-studied groups like Euglenophyta^49^ and Heterokontophyta - Coscinodiscophytina.^50^ Most of the studies we compiled from Heterokontophyta - Ochrophytina, the third most explored taxonomic group in MNP synthesis, were also focused on AuNPs.^67,79^ The particular traits of Au and AuNPs and their relatively lower exploration compared with Ag and AgNPs highlight the potential of gold to open new avenues for research, particularly in underexplored areas, making its continued use and investigation highly valuable.

Unlike Ag and Au, certain characteristics limit the use of many metals in nanoparticle synthesis, and even when MNPs are produced, they often have limited applications. For instance, metals like lead and cadmium are highly toxic, posing significant safety and environmental concerns,^138,139^ while rhodium and iridium are rare and prohibitively expensive.^140^ Despite these challenges, green synthesis has been reported for less common metals such as ruthenium,^141^ zirconium,^13^ and rubidium.^142^ Toxic metals have also been explored, with cyanobacteria and angiosperms being used to produce CdNPs.^143−146^ Remarkably, some organisms naturally process toxic metals into MNPs. For instance, the pteridophyte *Salvinia minimum* Baker can remove lead from water and synthesize PbNPs in its tissues through natural green synthesis.^147^ These examples underscore the diverse challenges and approaches in nanoparticle synthesis, emphasizing the need for exploring practical alternatives and expanding viable options, mainly considering that only 14.3% of the MNPs described in this review are formed by metals distinct from Ag, Au, Zn, and Cu.

### 4.2 Photoautotrophic Organisms Used in NP Green Synthesis

The large number of species and higher taxonomic groups, with diverse physiological characteristics, is in itself a reason for there being a wide range of green-synthesized NP types^44^ since this kind of synthesis is based on the natural metabolic activities of these organisms.^31^ However, our literature review showed that this biological potential is little explored, with more than 50% of the studies focused only on angiosperms, with a secondary relevancy for cyanobacteria and Heterokontophyta - Ochrophytina. The positive aspect is that these studies were focused on several species, making exploration within a taxonomic group more appropriate than if the focus was more restricted to a few species.

Since angiosperms are the most species-rich group of photosynthetic organisms,^45^ it is both expected and logical that many studies on the green synthesis of MNPs focus on these organisms. The structural complexity of plant tissues significantly contributes to their potential for synthesizing MNPs, as different plant parts exhibit distinctive biochemical characteristics.^148,149^ These parts, ranging from leaves and stems to roots, barks, fruits, and flowers, each provide unique secondary metabolites that play a key role in nanoparticle formation.^150^ Nearly all studies focusing on plants have concentrated on reproductive structures and leaves, indicating that many other plant parts remain underexplored.

More specifically on angiosperms, another advantage is the fact that these plants are used in many human activities, resulting in plant wastes, such as byproducts from food production and agricultural residues,^151,152^ that could be used for MNPs synthesis. Several studies have explored this approach by utilizing fruit and vegetable peels^153−155^ or sugar cane waste (bagasse)^156^ that would otherwise be discarded, transforming them into low-cost resources with minimal environmental impact for MNPs green synthesis. These methods are even improving the already sustainable aspect of the green synthesis of MNPs.

Cyanobacteria, with over 5700 described species,^46^ are easily cultivated and contain bioactive compounds that can serve as reducing and stabilizing agents in the synthesis of MNPs.^157,158^ These microorganisms can form blooms in response to nutrient inputs from anthropogenic sources, which often cause environmental problems, particularly in freshwater bodies.^159^ Cyanobacterial blooms can form dense, toxic surface mats, threatening ecosystems, water quality, and human health.^159^ Interestingly, some genera well-known for causing these blooms, such as *Microcystis* Lemmermann and *Dolichospermum* (Bornet & Flahault) P. Wacklin, L. Hoffmann & Komárek, have been shown to produce MNPs,^44,160^ offering an opportunity to turn this environmental challenge into a resource.

Like cyanobacteria, algae can also form blooms in response to nutrient influx, particularly macroalgae^161,162^ and dinophytes,^163^ though these events typically occur in marine environments. Seaweeds from the Chlorophyta, Rhodophyta, and Ochrophytina groups are utilized for MNP production, with genera like *Ulva*, *Gracilaria*, and *Sargassum* serving as prominent examples^64,65,83−85,164^ and commonly forming algal blooms.^161,162^ Harnessing the biomass from these blooms for nanoparticle production presents a valuable opportunity to address both environmental and economic concerns. Additionally, we emphasize that to date, there are no reports of NPs produced by dinophytes, and these organisms could represent a valuable resource for this field.

Although angiosperms and cyanobacteria are comparatively well studied for MNP synthesis, many photoautotroph groups have been largely neglected for no clear reason, since they are all diverse, commonly found in nature, and easy to culture. Some algal groups had only one study regarding NMP production, such as Euglenophyta^49^ and Heterokontophyta - Coscinodiscophytina.^50^ On the other hand, dinophytes, cryptophytes and haptophytes, which together comprise 3718 species,^46^ have yet to be explored for nanoparticle production.

Similar to the less studied groups of algae, bryophytes, the second most diverse group of plants,^45^ have been minimally studied regarding MNP production, leading to the underutilization of their vast research and application potential. These plants possess unique characteristics that make them excellent models for MNP production. Bryophytes are found on all continents,^165^ thriving in diverse environments ranging from humid tropical forests^166^ to rocky outcrops,^167^ and both hot^168^ and cold^169^ deserts. Additionally, they produce a wide array of secondary metabolites,^170,171^ which can act as reducing and stabilizing agents in the green synthesis of MNPs. Some species can even be cultivated in bioreactors,^172^ producing large quantities of biomass that could be harnessed for MNP production. Given their vast untapped potential, further research into bryophytes’ capabilities for nanoparticle synthesis could significantly expand available green synthesis methods and improve our understanding of their practical applications.

### 4.3 Characteristics of Monometallic NPs Green-Synthesized by Photoautotrophs: Does the Taxonomic Group Matter?

As size is one of the most important characteristics determining MNP activity,^173,174^ it is essential to search for organisms that produce smaller NPs or a variety of NP sizes (although always small) that could expand the spectrum of applications. Our results showed that among the two more investigated metals for green synthesis of NPs, Au could be more interesting than Ag, since AuNPs were significantly smaller and showed more size differences among taxonomic groups than AgNPs. Thus, directing research to search for a specific type of nanoparticle, considering the effects of major taxonomic groups, is also easier to accomplish with AuNPs. For instance, charophytes could be highlighted, as they produce some of the smallest AuNPs compared to other organisms. Although AgNPs can be green-synthesized with specific sizes depending on the photoautotroph species, no clear patterns were observed among major taxonomic groups, making it more challenging to guide research across the vast number of species.^44^ In summary, these findings about MNP sizes are essential for facilitate the selection of appropriate metals and organisms to achieve precise control over nanoparticle size for optimizing specific applications.

This review revealed that most photoautotrophs predominantly produce spherical NPs, except bryophytes, which showed a relevant percentage of articles reporting cubicNPs.^88^ The size and shape of NPs are closely linked to their activities, with specific forms offering advantages for particular applications.^174^ Thus, researchers could search for less common NPs because they can show better activities on some occasions. For example, cubic AgNPs demonstrated better antibacterial performances and were less problematic in toxicological tests than spherical AgNPs.^175^ The cubic shape is also valued because it is one of the best morphologies to form close-packed structures, forming more ordered architectures and also providing larger interfaces between the contacted NPs than other shapes.^175^ In conclusion, bryophytes are a group of special interest for the green-synthesis of very relevant MNPs based on their shape.

Regarding NP dispersity, the fact that the vast majority of studies do not mention this characteristic is a major flaw, as it makes it difficult to understand whether the NPs produced by these organisms are suitable for applications. Considering only the articles that mention the dispersity of NPs, it was possible to note that monodisperse (more uniformly disperse) MNPs were more commonly produced by practically all groups of photoautotrophs than polydisperse ones, which suggests that these organisms can really be a tool for the synthesis of MNPs of interest. This is a consequence of monodispersity producing more stable colloidal suspensions, with higher efficiency of MNPs for many processes and higher predictability in results.^10,11^ More specifically, we observed that more than 50% of the studies focused on macroalgae, Chlorophyta, and on Heterokontophyta - Ochrophytina, reported that the MNPs were monodisperse, suggesting a high likelihood of achieving consistent results with these organisms.

### 4.4 Why Should We Invest in Synthesizing Bimetal NPs Through Green Methods?

NPs composed of two different metals are named bimetallic NPs and exhibit enhanced catalytic, optical, electronic, and thermal properties compared to their monometallic counterparts, with their characteristics being influenced by factors such as size, composition, and synthesis methods.^176,177^ Bimetal NPs combine the unique properties of two different metals, which result in characteristics not found in the monometallic NPs.^178^ Thus, they hold significant untapped potential within the realm of green synthesis. They are commonly produced through conventional nanoparticle methods and have notable applications in the biomedical field.^179,180^ Despite their highly valuable characteristics, bimetallic NPs have not been the focus of research aimed at green synthesis using photoautotrophs, and our review indicates a great scarcity of these types of NPs.

The green synthesis of bimetallic NPs was restricted to a few groups of autotrophs, being only synthesized using Cyanobacteria, Chlorophyta, microalgae, and angiosperms. Additionally, the metal combinations have also been poorly explored. Given the vast diversity of photosynthetic organisms and metals available, the potential for the green synthesis of bimetal NPs is considerably high. The small number of studies was sufficient to show that bimetallic NPs have positive outcomes, particularly in biomedical applications.^113,118−120^ We believe that it is necessary to have more studies on these NPs, since they present significant opportunities, and their expanded application could drive innovative advancements in the field.

### 4.5 Applications of MNPs: Exploring Endless Possibilities

Among the most common uses for green-synthesized MNPs in biomedicine, electronics, industrial catalysis, cosmetics, and environmental technology,^31^ our data revealed that the most tested application of the MNPs synthesized with photoautotrophs was the antimicrobial capacity. Being the most commonly produced MNPs, the AgNPs stand out as the most widely used MNPs for this purpose, regardless of the taxonomic group used for their synthesis. Indeed, AgNPs derived from Cyanobacteria,^181^ Chlorophyta,^182^ Rhodophyta,^183^ Heterokontophyta,^74^ bryophytes,^88^ pteridophytes,^184^ gymnosperms,^96^ and angiosperms^185^ have all been documented to possess this antimicrobial capacity.

Gold NPs can also have relevant antimicrobial activity. They can have this property enhanced when conjugated with small molecules such as antibiotics, drugs, vaccines, and antibodies, a combination that is also more efficient than the use of these molecules alone.^186^ Green-synthesized AuNPs produced from many photosynthetic groups exhibit this capability, such as angiosperms^154^ and Heterokontophyta - Ochrophytina.^187^ The AuNPs described in the literature were very efficient for catalysis applications, being notably active under mild conditions, even at ambient temperatures or lower, which distinguishes them as exceptionally versatile and effective.^188,189^ In fact, we have compiled numerous studies investigating the potential of AuNPs in catalysis.

Many of the MNP applications highlight silver and gold as primary metals, but other metals can be crucial for some specific applications. For example, ZnNPs have been reported as effective adsorbents for positive metal ions in wastewater,^190,191^ and green-synthesized ZnNPs have already been tested for this purpose.^192^ This context underscores the distinctive potential of NPs and highlights the benefits of green synthesis methods. Embracing green synthesis not only promotes sustainability but also opens new avenues for developing effective and environmentally friendly solutions across various applications.

### 4.6 The Influence of Experimental Parameters in the Morphology of Green-Synthesized MNPs

Although our results highlight patterns regarding the autotrophs and their bioproducts used in the green synthesis of MNPs, other factors require a deeper exploration to fully understand their impact on MNP morphology. Many experimental parameters have been shown to significantly influence the stability, shape, and size of green-synthesized NPs, such as temperature,^193−195^ pH,^194,196,197^ extract concentration,^195,197,198^ concentrations of metal salts,^197,198^ and reaction time.^199^ For example, in the biosynthesis of gold NPs using angiosperm leaf extracts, higher extract concentrations and temperatures resulted in smaller, spherical MNPs, while lower concentrations and temperatures led to larger MNPs with diverse morphologies.^195^ Another example was an experiment in which was observed the synthesis of AuNPs utilizing angiosperm leaf extracts over a time span of 5–480 min, with the reaction medium reaching a plateau after 60 min, signifying the complete reduction of the gold precursor into gold nanoparticles.^199^ Therefore, understanding the interplay between these experimental parameters, along with considerations regarding the groups of organisms used, is crucial for optimizing the green synthesis of MNPs and guiding researchers to focus on specific factors to achieve NPs with the desired characteristics.

Additionally, the colloidal stability of MNPs can be significantly influenced by the medium in which they are dispersed, as the presence of specific ions or molecules can impact their size, dispersion, and overall stability.^200,201^ This factor is particularly crucial for a wide range of applications, with a stable dispersion in solution being essential for ensuring the NPs’ effective performance.^202,203^ Long-term stability is also a key consideration, particularly for commercially available NPs, since ensuring the stability of nanoparticles both in solution and in the solid state for prolonged periods of time is critical to their usability.^204,205^ Common dispersing media include water,^205,206^ culture media,^207^ and buffers,^207^ each of them having distinct effects on the NPs’ stability. Despite their importance, the stability of NPs in different media remains an unexplored area, and further research is essential to establish clear, standardized guidelines for selecting optimal conditions tailored to specific applications.

## 5 Conclusion

The comprehensive analysis of monometal NPs synthesized using extracts from photosynthetic organisms highlights notable trends in the diversity of both metals and organisms employed. Silver dominates as the most frequently used metal, followed by gold, zinc, and copper. Among the biological sources, angiosperms are the most commonly utilized group, reflecting their structural complexity and the availability of various plant parts for extract preparation. These patterns prompt us to explore organisms such as dinophytes, haptophytes, and cryptophytes as well as metals such as aluminum, bismuth, and titanium, which are often overlooked in nanoparticle production. Unique patterns emerge regarding the size and diversity of the NPs produced. While AgNP sizes show no significant variation across taxonomic groups, AuNPs exhibit differences, with Charophyta generating the smallest AuNPs. The smallest sizes for other metals, such as Zn and Cu, further illustrate that specific taxonomic groups can potentially influence nanoparticle dimensions due to differences in their biochemical composition. Bryophytes are recommended for the production of cubic MNPs. Overall, the findings underscore the substantial potential of photosynthetic organisms in the green synthesis of MNPs, with considerable variability in both the sources and the NPs’ properties. This variability provides opportunities to tailor nanoparticle synthesis for specific applications, leveraging the diversity of natural materials while emphasizing sustainable and eco-friendly methods.

## Supporting Information Available

All detailed information from the compiled articles, including species, metals used in the green synthesis of MNPs, and characteristics such as size, shape, and dispersity (XLSX)
